# Machine learning for atrial fibrillation risk prediction in patients with sleep apnea and coronary artery disease

**DOI:** 10.3389/fcvm.2022.1050409

**Published:** 2022-12-07

**Authors:** Carlos A. O. Silva, Carlos A. Morillo, Cristiano Leite-Castro, Rafael González-Otero, Michel Bessani, Rafael González, Julio C. Castellanos, Liliana Otero

**Affiliations:** ^1^Programa de Pós-graduação em Inovação Tecnológica, Universidade Federal de Minas Gerais, Belo Horizonte, Minas Gerais, Brazil; ^2^Department of Cardiac Sciences, Cumming School of Medicine, Libin Cardiovascular Institute, University of Calgary, Calgary, AB, Canada; ^3^Departamento de Engenharia Elétrica, Escola de Engenharia, Universidade Federal de Minas Gerais, Belo Horizonte, Minas Gerais, Brazil; ^4^Departamento de Economía, Facultad de Ciencias Económicas y Administrativas, Pontificia Universidad Javeriana, Bogotá, Colombia; ^5^Instituto del Corazón de Bucaramanga, Bogotá, Colombia; ^6^Departamento de Dirección General, Hospital Universitario San Ignacio, Bogotá, Colombia; ^7^Centro de Investigaciones Odontológicas, Facultad de Odontología, Pontificia Universidad Javeriana, Bogotá, Colombia

**Keywords:** atrial fibrillation, machine learning, risk prediction, survival analysis, sleep apnea, coronary artery disease

## Abstract

**Background:**

Patients with sleep apnea (SA) and coronary artery disease (CAD) are at higher risk of atrial fibrillation (AF) than the general population. Our objectives were: to evaluate the role of CAD and SA in determining AF risk through cluster and survival analysis, and to develop a risk model for predicting AF.

**Methods:**

Electronic medical record (EMR) database from 22,302 individuals including 10,202 individuals with AF, CAD, and SA, and 12,100 individuals without these diseases were analyzed using K-means clustering technique; k-nearest neighbor (kNN) algorithm and survival analysis. Age, sex, and diseases developed for each individual during 9 years were used for cluster and survival analysis.

**Results:**

The risk models for AF, CAD, and SA were identified with high accuracy and sensitivity (0.98). Cluster analysis showed that CAD and high blood pressure (HBP) are the most prevalent diseases in the AF group, HBP is the most prevalent disease in CAD; and HBP and CAD are the most prevalent diseases in the SA group. Survival analysis demonstrated that individuals with HBP, CAD, and SA had a 1.5-fold increased risk of developing AF [hazard ratio (HR): 1.49, 95% CI: 1.18–1.87, *p* = 0.0041; HR: 1.46, 95% CI: 1.09–1.96, *p* = 0.01; HR: 1.54, 95% CI: 1.22–1.94, *p* = 0.0039, respectively] and individuals with chronic kidney disease (CKD) developed AF approximately 50% earlier than patients without these comorbidities in a period of 7 years (HR: 3.36, 95% CI: 1.46–7.73, *p* = 0.0023). Comorbidities that contributed to develop AF earlier in females compared to males in the group of 50–64 years were HBP (HR: 3.75 95% CI: 1.08–13, *p* = 0.04) CAD and SA in the group of 60–75 years were (HR: 2.4 95% CI: 1.18–4.86, *p* = 0.02; HR: 2.51, 95% CI: 1.14–5.52, *p* = 0.02, respectively).

**Conclusion:**

Machine learning based algorithms demonstrated that CAD, SA, HBP, and CKD are significant risk factors for developing AF in a Latin–American population.

## Introduction

Atrial fibrillation (AF) is the most common heart rhythm disorder, however, frequently remains undiagnosed, or manifests sub-clinically. The prevalence of AF increases with age and is approximately 15% in individuals older than 80 years (1, 2). The worldwide increasing prevalence of AF may be explained by population aging and the increased prevalence of risk factors such as obesity (3). Additionally, undiagnosed obstructive sleep apnea (OSA) may contribute to the increasing incidence of AF and coronary artery disease (CAD) (4, 5). CAD is present in 17–46.5% of patients with AF (6, 7). Both AF and CAD share associated risk factors such as obesity, OSA, hypertension (HBP), diabetes mellitus (DM), family history, age, sex, ethnicity, sedentary lifestyle, smoking, heart failure, and valvular heart disease (8–10). Of note up to 30% of AF individuals may be asymptomatic, increasing the risk of stroke, and heart failure, and reducing overall survival, thereby incrementing healthcare costs (11).

Screening for detection of subclinical AF has been recommended by multiple cardiovascular and stroke guidelines. However, significant questions regarding the best technology and the duration of monitoring have been raised. Additionally, routine use of technology for AF detection is pragmatically limited (12). Incremental usage of wearable needs to be implemented to increase detection design tools for risk stratification, unfortunately healthcare systems are conservative and delayed in integrating these technologies as a population-based measure (13). Effective detection of subclinical AF may be enhanced by risk-prediction models developed through machine learning methods used in precision medicine (14).

Electronic medical records (EMR) are a valuable source for research since predictive variables can be extracted to develop these models. EMR are being used to classify, diagnose and predict future hospitalization through machine learning methods (15, 16). Lima et al. state that machine learning aims to study and develop computational methods to obtain systems capable of acquiring knowledge automatically (17). The construction of this occurs with the listing of input and output variables from sampled data. The automatic variable selection included in machine learning techniques could reduce the assumptions and human involvement required in other prognostic models (18).

Machine learning methods can reduce bias caused by human intervention resulting in more accurate prognostic models and have been useful to establish clinical phenotyping, risk stratification and treatment outcomes (19, 20). However, approximately 10% of AF patients may have been misclassified with AF algorithms and the risk factor profile over time should be considered in the development of these algorithms (21). The Multi-ethnic study of atherosclerosis (MESA) has employed machine-learning techniques to predict 5-year AF risk by adding novel candidate variables identified by machine learning and derived from the Cohorts for Heart and Aging Research in Genomic Epidemiology (CHARGE) AF Enriched score (22). However, subclinical AF was not identified, but this limitation is present in other AF prediction studies (23). Additionally, AF risk prediction models may be limited by racial/ethnic diversity. Therefore, the prevalence of AF varies among different ethnic populations, furthermore, findings from previous studies are inconsistent. AF seems less prevalent in individuals of Asian and African ethnicities compared to Caucasian and Hispanic individuals, but other investigations have reported a higher prevalence of AF in African Americans compared to Caucasians (24–26).

Predisposing factors for AF include biologic and genetic factors that include ionic K^+^ channel alterations that reduce the atrial refractory period and increase dispersion of refractoriness promoting re-entry while decreasing automaticity (9, 27), and genes and micro-RNA that control the ion regulating activity of Ca^2+^ and K^+^ channels that seem to be involved in atrial myopathy promoting AF (28). Cardiometabolic risk factor such as DM, HBP, obesity, OSA, and CAD are highly prevalent in AF. Therefore, genetic and biological mechanisms associated with these risk factors should be considered when developing AF risk prediction models.

Obstructive sleep apnea has been associated with several cardiovascular risk factors, including hypertension, AF, CAD, and cardiovascular mortality. However, OSA is often underestimated in cardiovascular practice (5). However, the causal effect on an increased risk of cardiovascular disease (CVD) has not been clearly established (29). Implementing artificial intelligence in large data samples of patients with OSA, AF, and CAD may clarify the cause effect role of these risk factors.

Given the aforementioned issues, the role of OSA and CAD in AF risk is still unclear. This study employs exploratory data analysis and machine learning techniques to develop a risk model for predicting AF and to evaluate the role of CAD and OSA in determining AF risk.

## Materials and methods

### Population

This is a retrospective cohort study, that used data derived from EMR from 22,302 individuals aged > 18 years of age who were seen at the Instituto del Corazón de Bucaramanga/Bogotá in Colombia, during 2010–2019. Data were de-identified, removed the duplicated and organized prior to analysis. The study was approved by the ethics committee at the Pontificia Universidad Javeriana (OD-0249). The sample included a database from 22,302 individuals (10,202 individuals with AF, CAD, and OSA and 12,100 individuals without AF, CAD, and OSA). The total of records (medical entities per visit) of these individuals during 2010–2019 were 177,656 (74,759 records of individuals with AF, CAD, and OSA and 102,897 records of individuals without AF, CAD, and OSA) (Table 1). The information contained in the records involved: date of consultation, diagnosis, international code of diagnosed disease (ICD), age, sex, height, weight, body mass index (BMI), symptoms, time until the diagnosis of CAD, AF, or OSA; results of cardiovascular test and procedures, family history, pharmacological, toxic, surgery, allergies and pathologic history, among others. The international classification of diseases 10th revision procedure classification system (ICD-10-PCS) used the ICD up to the categorical level (three digits, i.e., Z99) to assign the comorbidities in each patient. The group of patients with CAD included individuals who had obstruction of at least one main coronary artery of 50% or more, diagnosed by coronary angiography. All patients with OSA had been diagnosed through polysomnography (apnea-hypopnea index of five or more events per hour and oxygen desaturation of 3% or more). The diagnosis of AF was confirmed by 12 lead ECG. For patients diagnosed with the diseases of interest (CAD, OSA, and AF), all available data up to the date of consultation of the first diagnosis of the disease was used; for others, all available history.

**TABLE 1:** Distribution of sample.

Description	Total of individuals
Cohort of patients with AF	1,686
Cohort of patients with CAD	7,879
Cohort of patients with OSA	1,032
Cohort of patients with OSA and CAD	530
Cohort of patients with OSA and AF	172
Cohort of patients with AF and CAD	726
Cohort of patients with SA, AF, and CAD	75
Cohort of patients with AF, OSA, and CAD	10,202 (74,759 records)
Cohort of patients with other diseases	12,100 (102,897 records)

Total of individuals in electronic medical record (EMR) classified by groups of disease. AF, atrial fibrillation; CAD, coronary artery disease; OSA, obstructive sleep apnea.

### Data analysis

Electronic medical record information was cleaned to process data with well-defined criteria and context and patients with missing data were removed. Only data until the first diagnosis of the disease of interest is considered. First, the k-nearest neighbor (kNN) algorithm was applied to classify patients into groups to perform risk models for AF, CAD, or OSA. Next, the elbow method was applied to the data to identify the optimal number of groups in the data. The k-means method was used to group the sample in clusters before the event of interest (AF, OSA, or CAD) occurred. In the final step, survival analysis was performed using Cox proportional hazards (PH) models to evaluate the relationship between comorbidities and time to develop the diseases AF, CAD, or OSA.

### Machine learning

#### Clustering

The experiment aims to identify clusters for AF, CAD, and OSA. The variables selected to perform the cluster for each disease included sex, age, and the time (in days) until the diagnosis of AF, CAD, or OSA. The Elbow method was applied to identify the ideal number of groups. The K-means clustering technique was used to perform the clustering.

#### Survival analysis

Survival analysis using multivariable Cox proportional hazard and Kaplan–Meier models was performed to identify the risk and time involved until the development of AF, OSA, or CAD. The start condition was age ≥ 18 years, and the time until diagnostic (T variable) is more than 0. The metrics used to measure the risk was hazard ratio (HR), and the model concordance was C-index.

The survival data for the individual i (*i* = 1,⋯, *n*) in the study, are represented, in general, by the pair (t_*i*_, δ_*i*_) where t_*i*_ is the time of breakdown and δ_*i*_ is an indicator variable of breakdown. In the presence of co-variables measured at the same individual level, such as xi = (sex, age, BMI), the data are represented by (t_*i*_, δ_*i*_, x_*i*_). There is no special case of interval data, yet there is a representation (l_*i*_, u_*i*_, δ_*i*_, x_*i*_) where l_*i*_ and ui are, respectively, the lower and upper limits of the comparison range for the *i*-th individual.

The survival function is used to represent the probability of the event of interest, i.e., patient survival, during an interval of time *t*. In mathematics’ notation, we have: *S(t) = P(T ≥ t)*, and the cumulative distribution function represents the probability of no survival on time *t* is *F*(*t*) = 1−*S*(*t*) and the density function of no survival can be obtained by $f\,\left(t\right)=\frac{d}{d\,t}\,F\,\left(t\right)$, to continuous cases, and $f\,\left(t\right)=\frac{\left[F\,\left(t+\Delta \,t\right)-F\,\left(t\right)\right]}{\Delta \,t}$, where Δt denote a time interval to discrete cases.

The formula used for Cox PH model was: *h*(*t*) = *h*_0_(*t*) ⋅ *g*(β*X*′).

where *g* is a function such that *g*(0) = 1.

Therefore, Cox proportional-hazards analysis with a time-dependent definition for the AF, CAD, or OSA apparition were used (30). HR = exp[sum(Beta_n*Xn′)]/exp[sum (Beta_n*Xn″)]. Xn′ = control group. Xn″ = study group. HR > 1: increased risk to develop earlier the interest condition.

#### Risk algorithms

The objective of conducting this algorithm is to check if it is possible to identify the development of disease *Y* = {AF, CAD, OSA} given the patient was diagnosed according to an ICD code *X*, where *X* is derived from the EMR and the kNN method applied. Machine learning through kNN was employed to identify the risk of developing AF, OSA, or CAD, based only on EMR and without the information of the diagnostic. To build prognostic models by machine learning, the parameter K varied between 3 and 30. For each one of these values, it carried out ten experiments and, in each experiment, the sample was randomly split into a training cohort (70% of the patients), and a validation cohort (30% of patients) (31). The validation cohort was used to evaluate the effectiveness of the final models.

## Results

The present analysis included 10,202 individuals represented by 1,686 with AF, 7,879 with CAD, and 1,032 with OSA. The comparison group include 12,100 individuals without these diseases. The prevalence of AF, OSA, and CAD in groups classified by sex and age are shown in Table 2.

**TABLE 2:** Prevalence of AF, OSA, and CAD in groups classified by sex and age.

Description	*% Male				*% Female				Number of individuals		
			
	18–40 years	41–55 years	56–75 years	76–95 years	18–40 years	41–55 years	56–75 years	76–95 years	Male	Female	Male + female
OSA	4.32	15.49	61.25	18.92	2.77	14.95	59.27	22.99	671	361	1,032
CAD	0.89	10.27	58.64	30.18	0.96	7.65	52.48	38.89	5,907	1,972	7,879
AF	2.01	7.76	46.14	43.85	1.67	6.04	41.61	50.67	1,090	596	1,686
OSA + CAD	1.86	14.93	63.46	19.73	0	9.67	64.51	25.80	375	155	530
OSA + AF	4.31	16.37	62.06	17.24	0	3.57	39.28	57.14	116	56	172
AF + CAD	0.39	5.56	46.35	47.72	0.45	3.16	38	58.37	505	221	726
OSA + AF + CAD	2.04	18.36	61.22	18.36	0	0	30.76	69.23	49	26	75

All diseases were more prevalent in males between 56 and 75 years old, while in females AF was more frequent in the group older than 75 years of age. *% Percentage into male or female group by disease. AF, atrial fibrillation; CAD, coronary artery disease; OSA, obstructive sleep apnea.

### Cluster analysis

**FIGURE 1:**
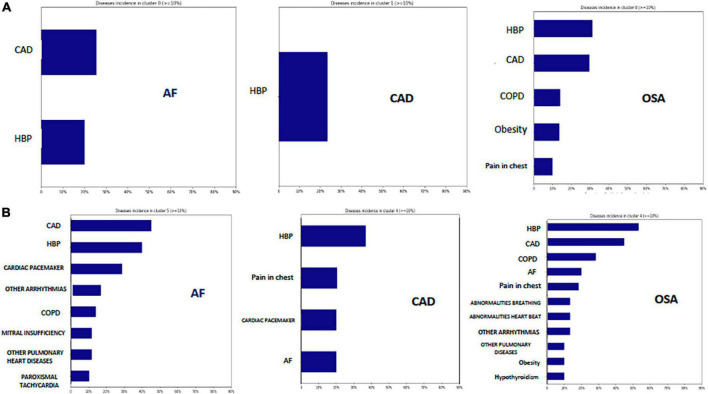
Clusters. **(A)** Clusters with the major number of individuals (66–91.5%) for AF, CAD, and OSA. CAD and HPB were the most prevalent diseases in AF cluster, HPB was the most prevalent diseases in CAD cluster, and HBP and CAD were the most prevalent diseases in the OSA cluster. **(B)** Show the same prevalent diseases for each cluster (4.6–16% of individuals), but with more comorbidities in each group. CAD, Coronary artery disease; HBP, High blood pressure; COPD, Chronic obstructive pulmonary disease.

Seven clusters were identified for each disease AF, CAD, and OSA. The clusters that grouped the majority number of patients are described in Table 3 and Figure 1. The most prevalent diseases in the first cluster that group the largest number of individuals with AF (79.2%) were CAD (25.57%) and HBP (20.01%), in the second cluster of AF (9.14% of individuals) CAD and HBP were also the most prevalent diseases, but other diseases such as chronic obstructive pulmonary disease (COPD) were included in this cluster. The most prevalent disease in the first cluster that group the largest number of individuals with CAD (91.5% of individuals) was HBP (23.39%), but 4% of these individuals had AF. The most prevalent diseases for the first cluster (66.76% of individuals) second cluster (16.08%) that group the largest number individuals with OSA were HBP, CAD, and AF.

**TABLE 3:** Clustering.

	Cluster 1	Cluster 2	Cluster 1	Cluster 2	Cluster 1	Cluster 2
Description	AF	AF	CAD	CAD	OSA	OSA
Mean of age	68.16	70.31	64.53	65.21	60.89	65.03
Total male %	61.18	63.7	75.94	71.06	61.44	65
Total female %	38.82	36.3	24.06	28.94	38.56	35
HBP %	20.01*	40*	23.39**	36.79**	31.32***	53.33***
CAD %	25.57*	45.18*	–	–	29.71***	45***
AF %	–	–	4	2.12	8.43***	20***
OSA %	1.36	1.48	0.74	2.83	–	–
Chronic kidney disease %	2.3	3.70	2.3	5.66	0	0
Hypertensive heart disease %	2.92	6.66	2.9	8.8	4.81	3.33
Cardiac pacemaker %	9.23	28.8	4.77	20.12	3.61	3.03
Mitral valve insufficiency %	5.13	11.85	15**	7.86**	0.4	3.33
Obesity %	1.02	2.96	2.01	2.2	13.65	10
Total sample %	79.2	9.14	91.5	4.69	66.75	16.08

The two clusters with the largest number of patients for AF, CAD, and OSA are included in the table. The mean of age, the percentage of male and female, and the frequency for the most prevalent disease for each cluster are reported. Although AF, CAD, and OSA were most prevalent in male, the percentage of female affected in each cluster was high. The most prevalent diseases in AF* clusters were CAD and HBP; in CAD** clusters were HBP and mitral valve insufficiency; and in OSA*** clusters were HBP, CAD, and AF.

### Survival analysis

**FIGURE 2:**
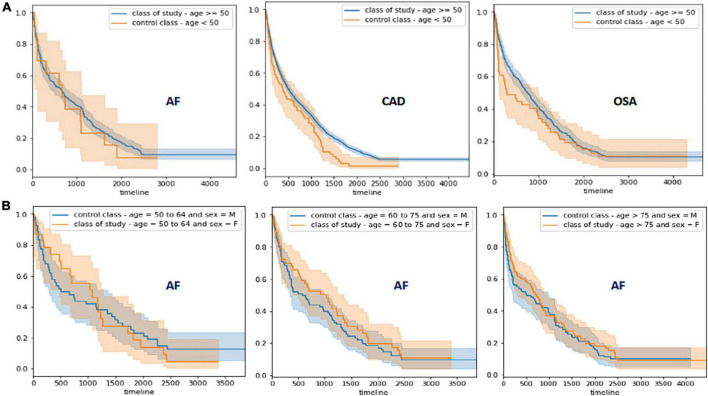
Survival Analysis for AF, CAD, and OSA. **(A)** Survival analysis for AF, CAD, and SA. The blue line represents study cohort and orange line the control cohort. The shading areas (blue and orange) represent the confidence intervals for each cohort. **(B)** Survival analysis for risk of AF development comparing male and females by decades of life.

The findings of survival analysis are shown in Figure 2 and Table 4. This analysis demonstrated that HBP, CAD, chronic kidney disease (CKD), and OSA significantly contribute to the AF outcome in patients older than 50 years (HBP = HR: 1.54, 95% CI: 1.22–1.94, *p* = 0.0039; CAD = HR: 1.49, 95% CI: 1.18–1.87, *p* = 0.0041; CKD = HR: 3.36, 95% CI: 1.46–7.73, *p* = 0.0023 and OSA = HR: 1.46 95% CI: 1.09–1.96, *p* = 0.01). Subsequently, patients with CAD develop AF earlier when CAD is associated with CKD (HR: 2.87, 95% CI: 1.06–7.81, *p* = 0.04). The survival analysis for the risk of AF comparing men and women by decades in patients older than 50 years showed that women develop AF earlier between 50 and 64 years when associated with HBP (HR: 3.75, 95% CI: 1.08–13, *p* = 0.04), in patients between 60 and 75 years with CAD (HR: 2.4, 95% CI: 1.18–4.86, *p* = 0.02) and OSA (HR: 2.51, 95% CI: 1.14–5.52, *p* = 0.02), and in women older than 75 years with HBP (HR: 2.1, 95% CI: 1.26–3.52, *p* = 0.0037) and CAD (HR: 1.67, 95% CI: 1.0–2.8, *p* = 0.05).

**TABLE 4:** Results of Cox proportional hazards (PH) model.

Variable	AF (*p*)	AF (HR)	AF (CI)	CAD (*p*)	CAD (HR)	CAD (CI)
Age	0.11	1.01	1.00–1.02	0.42	1.00	0.99–1.01
Sex	0.47	0.92	0.73–1.16	0.35	0.90	0.71–1.13
HBP	0.0039	1.54	1.22–1.94	0.01	1.28	1.06–1.54
AF	–	–	–	0.45	1.14	0.82–1.58
CAD	0.0041	1.49	1.18–1.87	–	–	–
SA	0.01	1.46	1.09–1.96	0.27	1.18	0.88–1.57
Chronic kidney disease	0.0023	3.36	1.46–7.73	0.15	1.39	0.89–2.16
Hypertensive heart disease	0.33	1.25	0.80–1.97	0.01	1.57	1.10–2.23
Cardiac pacemaker	0.05	1.46	1.00–2.14	0.03	1.61	1.06–2.46
Mitral valve insufficiency	0.72	1.07	0.76–1.50	0.24	1.38	0.81–2.37
Obesity	0.71	1.09	0.68–1.74	0.98	1.01	0.62–1.65

Table shows the results for survival analysis for AF and CAD. The results for risk of OSA were not significant (*p* < 0.05) for any comorbidity. HBP, CAD, OSA, and CKD were the risk factors to develop AF. HBP, hypertensive heart disease and having cardiac pacemaker were the risk factors for CAD. HR, hazard ratio; 95% CI, confidence interval.

### Risk algorithms

**FIGURE 3:**
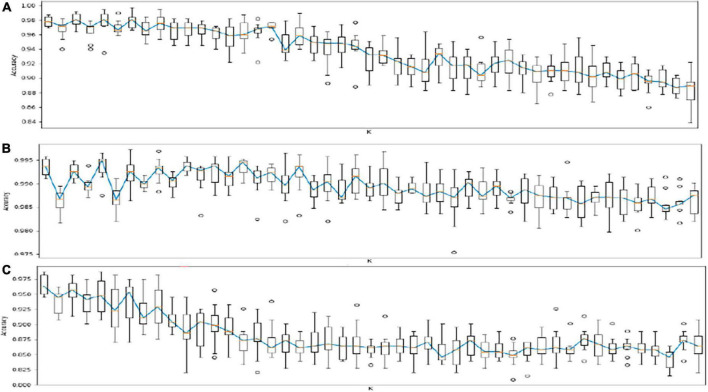
Results of risk algorithms through kNN analyses. Each algorithm of KNN analysis was tested 10 times in the phase or training (70%) and validation (30%). **(A)** Risk models for AF in individuals with CAD (ACC: 0.93; AUC: 0.81; Sensitivity: 0.63; and Specificity: 0.99). **(B)** Risk models for AF in individuals with OSA (ACC: 0.92; AUC: 0.70; Sensitivity: 0.99; and Specificity: 0.40). **(C)** Risk models for AF in all individuals of the sample (ACC: 0.95; AUC: 0.80; Sensitivity: 0.99; and Specificity: 0.63). AF, Atrial Fibrillation; CAD, Coronary Artery Disease; SA, Sleep Apnea; ACC, Accuracy; AUC, Area Under de ROC: The average value of sensitivity for all possible values of specificity; ROC, Receiver Operating Characteristic (ROC) Curve. A plot of test sensitivity as the y coordinate versus its 1-specificity or false positive rate (FPR) as the x coordinate.

The results of the risk models for AF in individuals with CAD and OSA are shown in Figure 3. These algorithms identified with high accuracy and sensitivity AF in individuals with CAD (Accuracy (ACC): 0.93; Area under the receiver operating characteristic curve (AUC): 0.81; Sensitivity: 0.63; Specificity: 0.99), in individuals with OSA (ACC: 0.92; AUC: 0.70; Sensitivity: 0.99; Specificity: 0.40), and in all individuals of the sample (ACC: 0.95; AUC: 0.80; Sensitivity: 0.99; Specificity: 0.63).

## Discussion

The present investigation applied conventional statistical and machine learning techniques on a cohort of 22,302 EMR records from Latin American patients to build risk models and determine the effect of demographic and comorbid predictors on AF during a follow-up of approximately 7 years. The relationship with CAD and OSA was also explored; the main findings were: (1) Cluster analysis confirmed that comorbidities associated with AF, CAD, and OSA were HBP, CKD, hypertensive heart disease, mitral regurgitation, having a cardiac pacemaker and obesity; (2) kNN Machine learning was useful to classify each disease associated to its comorbidities independently *via* clusters (AF, CAD, and OSA) with very high rates (> 95%) of sensitivity, specificity, AUC and ACC; (3) On average a 1.5-fold increase in developing AF was observed in individuals with HBP, CAD, and OSA and a threefold increase with CKD. AF was developed approximately 50% earlier than patients without these comorbidities in a period of 7 years (the median time to develop AF was 943 days); (4) Individuals with CAD and CKD develop AF significantly earlier compared to those with CAD and preserved kidney function; (5) Finally, women show a risk biggest of up to 67% to develop AF in a period of 7 years compared to when HBP and CAD are present, this finding was primarily related to age.

The most frequent comorbidities grouped in clustering were explored in the survival analysis to identify the role of CAD and OSA to determine the risk of developing AF, but also, the relationship between AF, CAD, and OSA with other comorbidities. As expected, the prevalence of AF, OSA, and CAD increased in individuals older than 50 years, therefore patients were divided into a study and control cohort accordingly. Three findings derived from the survival analysis were relevant: (1) the inflection time to evaluate the risk of AF is clearly 50 years and HBP, CAD, OSA, and CKD were significantly associated with AF risk within a time frame of almost 7 years (2,500 days), (2) comorbidities significantly associated with CAD risk in patients over 50 years old during the same time frame were HBP, hypertensive heart disease and having a pacemaker previously implanted. Finally, the lifetime risk to develop AF in women was the 7th decade, however there is an almost twofold increase in the risk of developing AF when associated HBP and CAD. The pathophysiologic mechanisms involved in the differential risk for men and women per decade are unclear and may include increased pregnancies due to repeated hormonal exposure and other metabolic factors (32); including earlier age at menopause associated with the anti-inflammatory effects of estrogens (33), DM has been associated with incident AF in women but not in men (34), and OSA is more frequent in women older than 55 years (35). Siddiqi et al. reported that women are at higher risk for incident AF than men when BMI is analyzed in stratified models (36). Although the accumulation of comorbidities increases the chance of developing risk factors, some factors such as family history of AF, ethnicity and genetic risk profile should be considered to explain the increased AF risk in older women (37). Future directions for research include artificial intelligence and precision medicine to prevent the higher risk of heart failure and stroke associated with AF in women (38). Biomarkers based on genetic studies may allow us to clarify our understanding of sex dimorphism in AF.

The relationship between AF and CAD may be explained by the following facts: AF and CAD share multiple comorbidities, and the most significant comorbidity for AF risk was HBP followed by CAD while the most significant comorbidities for CAD risk were HBP and Hypertensive heart disease. This two-way relationship between AF and CAD shares a common pathway of inflammation and co-existent risk factors. Similarly, AF is present in 17–47% of patients with CAD while the prevalence of CAD in patients with AF has been reported from 0.2 to 5% (39). AF has worse clinical outcomes in patients with preserved ejection fraction without CAD compared to those with CAD (40). Further studies are necessary to investigate the biological mechanisms involved in the AF and CAD relationship.

Obstructive sleep apnea increased by one and a half fold the risk of developing AF. This finding is in keeping with previous studies. OSA, is a sleep disorder that has been recognized as a risk factor for CAD and more recently for AF reportedly having a fourfold risk of developing AF compared to non-SA patients (41). However, the prevalence of OSA in patients with AF has been reported in few studies and OSA screening in patients with AF remains uncommon. Nevertheless, the prevalence of OSA reported for patients with AF fluctuates from 18 to 70% depending on diagnostic criteria, sex and altitude (42–45).

The results of our cluster analyses identified seven phenotypes for each group of study (AF, CAD, OSA) and showed that HBP was the most prevalent comorbidity associated to CAD, OSA, and AF. Cluster analytic techniques used in several studies have proposed phenotypic groups for CVD risk and include HBP, AF, CAD and renal disfunction as comorbidities with the highest risk for heart failure and death (46). The role of OSA in the phenotypes groups of CVD risk has not been extensively studied. It has been reported that cardiovascular risk among patients with OSA is related with excessively manifestation of the sleepy phenotype (47). The American heart association recommends screening for OSA in patients with poorly controlled hypertension, pulmonary hypertension, and recurrent AF (5).

Our kNN analysis indicates that an effective risk prediction model for AF derived from EMR derived comorbidities is feasible. Considering the increasing prevalence of AF in the population it is necessary to maximize the detection of AF cases and a potentially cost-effective method may be machine learning methods and artificial intelligence to appropriately apply precision medicine for diagnosis and personalized treatment. Hill et al. (48) evaluated statistical and machine learning models such as support vector machines, neural networks (NN), selector operator [Least absolute shrinkage and selection operator (LASSO)], random forests, Cox regression, and validated risk scores such as Framingham, Atherosclerosis Risk in Communities (ARIC) and CHARGE-AF to develop a risk prediction model to identify AF. They reported that one of the most specific risk models for AF is CHARGE-AF with 61% specificity compared with 52% using logistic regression. However, these risk models simulate linear relationships between covariates. In their risk model developed through machine learning, the model identified highly non-linear associations between covariates and incidence with 74.9% specificity and 75% sensitivity. This model was derived from previous risk models including CHARGE-AF, Framingham and ARIC and included demographic data, heart failure, DM, left ventricular hypertrophy, CAD, antihypertensive treatment and history of smoking among others (49–51). Nonetheless, this model was built using a UK population and did not consider OSA. In our study, the kNN algorithm for AF involved patients from one country in Latin America including OSA with Sensitivity: 1 and Specificity: 0.94. Our algorithm needs to be validated within other populations.

Other studies have reported the use of machine learning for the identification of patients with AF derived from physical examination findings or documented rhythm alterations detected by a smartwatch. Lown et al. (52) designed a wearable heart rate monitor and machine learning algorithm for AF detection and demonstrated a high accuracy to confirm AF with this design. Attia et al. (53) developed an algorithm to identify AF using artificial intelligence to detect the electrocardiographic signature of AF in normal sinus rhythm, Kwon et al. (54) used deep learning algorithms to detect AF through photoplethysmographic recordings and several other similar studies that employ machine learning and deep learning to identify AF in subclinical patients have been reported (55–57). None of these studies developed a machine learning algorithm to predict AF based on age, gender, or risk factors.

Incorporating machine learning systems to EMR for AF may be useful to determine the behavior of physiological data and the temporal relationships associated with risk factors. Cox proportional hazard regression and survival analysis have been employed to predict the response of pharmacological and electrical cardioversion therapies for AF (58, 59), however there are no reports that apply these machine learning techniques to identify AF risk factors. Our study is the first study to report the use of machine learning and survival analysis to develop clusters and risk models for AF in a Latin–American population.

## Limitations

Some limitations should be considered; EMR had to undergo a significant data cleaning process prior to analysis, similarly we cannot rule out that a significant proportion of patients over 65 years may have had subclinical AF and therefore not identified in this study. Finally, patients diagnosed in a secondary care clinic may have more comorbidities than patients in primary care or in the general population. Our study focused on comorbidities as risk factors for AF and future studies should include genomic, socioeconomic status, and family history for model risks.

## Conclusion

Machine learning identified risk factors for AF and other comorbidities in a large cohort of EMR derived from Latin American patients. Future prospective studies based on machine learning methods should be performed and include phenotype and genotype risk variables and comparing different populations. The identification of risk factors associated with AF may potentially provide better therapeutic results and tools for prevention policies in public health.

## Data availability statement

The raw data supporting the conclusions of this article will be made available by the authors, without undue reservation.

## Ethics statement

The studies involving human participants were reviewed and approved by the Ethics Committee at the Pontificia Universidad Javeriana (OD-0249). The patients/participants provided their written informed consent to participate in this study.

## Author contributions

CS performed the machine learning analysis, clustering, risk prediction, and survival analysis. CL-C, MB, and RG-O assisted in the data collection and supervision of the machine learning analysis. LO, CS, CL-C, MB, RG-O, and CM conceived and designed the study. CM, JC, RG, RG-O, and LO reviewed and edited the manuscript. All authors contributed to the article and approved the submitted version.
